# Archaic mitochondrial DNA inserts in modern day nuclear genomes

**DOI:** 10.1186/s12864-019-6392-8

**Published:** 2019-12-26

**Authors:** Robert Bücking, Murray P Cox, Georgi Hudjashov, Lauri Saag, Herawati Sudoyo, Mark Stoneking

**Affiliations:** 1 Department of Evolutionary Genetics, Max Planck Institute for Evolutionary Anthropology, Deutscher Platz 6, Leipzig, D04103 Germany; 2 School of Fundamental Sciences, Massey University, Palmerston North, 4442 New Zealand; 3 Institute of Genomics, University of Tartu, Tartu, 51010 Estonia; 4 Genome Diversity and Diseases Laboratory, Eijkman Institute for Molecular Biology, Jakarta, 10430 Indonesia; 5 Department of Medical Biology, Faculty of Medicine, University of Indonesia, Jakarta, 10430 Indonesia; 6 Sydney Medical School, University of Sydney, Sydney, NSW 2006 Australia

**Keywords:** Archaic introgression, NUMTs, Denisovans

## Abstract

**Background:**

Traces of interbreeding of Neanderthals and Denisovans with modern humans in the form of archaic DNA have been detected in the genomes of present-day human populations outside sub-Saharan Africa. Up to now, only nuclear archaic DNA has been detected in modern humans; we therefore attempted to identify archaic mitochondrial DNA (mtDNA) residing in modern human nuclear genomes as nuclear inserts of mitochondrial DNA (NUMTs).

**Results:**

We analysed 221 high-coverage genomes from Oceania and Indonesia using an approach which identifies reads that map both to the nuclear and mitochondrial DNA. We then classified reads according to the source of the mtDNA, and found one NUMT of Denisovan mtDNA origin, present in 15 analysed genomes; analysis of the flanking region suggests that this insertion is more likely to have happened in a Denisovan individual and introgressed into modern humans with the Denisovan nuclear DNA, rather than in a descendant of a Denisovan female and a modern human male.

**Conclusions:**

Here we present our pipeline for detecting introgressed NUMTs in next generation sequencing data that can be used on genomes sequenced in the future. Further discovery of such archaic NUMTs in modern humans can be used to detect interbreeding between archaic and modern humans and can reveal new insights into the nature of such interbreeding events.

## Background

The presence of mitochondrial DNA (mtDNA) sequences in nuclear genomes has been widely reported in various eukaryotic organisms [1, 2]. The transfer of such genetic material in humans, reported to still be an ongoing evolutionary process [3–6], has resulted in various fixed and polymorphic Nuclear Mitochondrial DNA segments (NUMTs) in present day genomes. In the human reference genome, a total of 755 NUMTs have been identified [7]. In addition to these NUMTs, many more polymorphic NUMTs have been detected in various human populations around the world [8] and the analysis of additional populations is expected to reveal many more polymorphic NUMTs. NUMTs vary in size and can consist of almost the entire mitochondrial genome. There is no evidence for a preference for the insertion of particular regions of the mtDNA genome, which is entirely represented by the various NUMTs in the nuclear genome [8, 9]. In general, NUMTs behave as noncoding sequences in the nuclear genome and evolve without any functional constraints [10]. Old insertions tend to get modified by deletions, duplications, inversions and other mutations over a long period of time until they are no longer recognizable as mtDNA. More recent insertions tend to still preserve the sequence at the time of their insertion due to the lower mutation rate in nuclear than in mitochondrial DNA (as reviewed in [11]). NUMT sequences have been used to reveal processes of molecular evolution in the absence of selection [12] and to date events of species divergence [13]. Moreover, their frequencies and presence-absence patterns have also been used to study the genetic relationships of different human populations [14–16]. NUMTs have also been used to detect admixture and hybridisation events between present or extinct species. Interbreeding events in insects [17] and ancient hybridisation between two monkey genera [18] have been detected by combining presence-absence analyses with information about the NUMT sequence. While NUMTs are usually transmitted vertically across generations and thus represent an ancestral state of the mtDNA of that species, they can also be transferred horizontally through interbreeding between different species or diverged populations. A NUMT arising via such interbreeding would not follow the mtDNA phylogeny of the individual it was found in. If the NUMT sequence is more similar to the mtDNA of another species or distant population, it would indicate introgression through interbreeding between these two lineages. In this study, we screened modern human genomes for fragments of mtDNA in the nuclear genome with a different phylogeny than that of modern humans. Such NUMTs can be used to detect and analyse interbreeding events in the evolutionary history of humans. It is already well-established that the ancestors of various modern human populations interbred with Neanderthals and Denisovans [19–22]. All genomes outside of sub-Saharan Africa contain ∼ 2% Neanderthal DNA [22], while Denisovan ancestry is less evenly distributed around the world; Eastern Eurasian and Native American populations only contain small amounts, whereas Oceanian populations derive up to ∼ 4% of their genome from Denisovan DNA [21, 23]. More recent studies have shown that interbreeding events between archaic and modern humans occurred several times during the evolutionary history of modern humans [24–28].

**Fig. 1:**
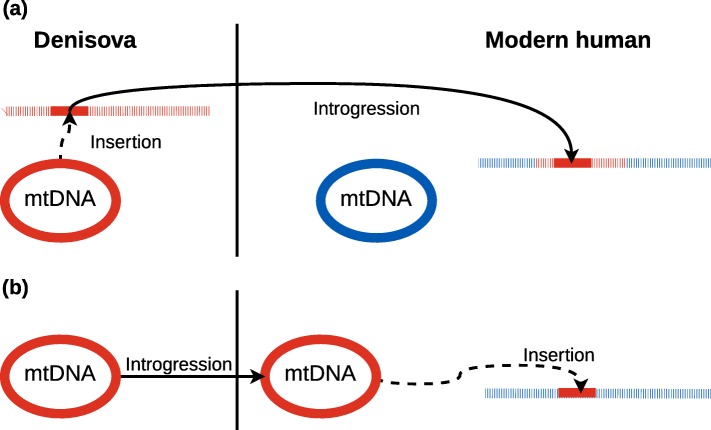
Hypotheses for NUMT introgression. Two possible scenarios for the introgression of Denisovan mitochondrial DNA (mtDNA) in modern human nuclear DNA. **a** The NUMT occurred in a Denisovan and introgressed together with its nuclear DNA. This path would result in a flanking region with Denisovan ancestry. **b** The mtDNA introgressed into a modern human, where it subsequently inserted into the nuclear DNA. mtDNA: solid colours; nuclear DNA: striped; Denisovan DNA: red; modern human DNA: blue

While the introgression of archaic nuclear DNA into modern humans has been widely detected, archaic mtDNA genomes have not been detected in modern humans. However, archaic NUMTs in modern humans have not been systematically investigated. There are two pathways by which archaic NUMTs could be introduced into modern human genomes (Fig. 1): (a) the NUMT arose in the archaic humans and was then transferred to modern humans along with other archaic nuclear DNA via interbreeding; (b) the NUMT arose *de novo* in the germ line of an archaic-modern human hybrid with an archaic mtDNA genome, and was subsequently passed on to the modern human population. This latter pathway is perhaps of more interest as it provides information about the sex of the interbreeding individuals that is otherwise not available from the archaic nuclear DNA in modern humans. In this case, it would indicate that the original interbreeding involved an archaic female and a modern human male. These two pathways can be distinguished by examining the genomic region surrounding the archaic NUMT: if the archaic NUMT had occurred in an archaic human, then the surrounding genomic region should consist of archaic DNA; if the archaic NUMT arose *de novo* in an archaic-modern human hybrid, then the surrounding genomic region should consist of modern human DNA (unless the NUMT happened to insert into a region of archaic DNA in the hybrid).

To detect such NUMTs, we scanned 221 genomes from Indonesia and Oceania for NUMTs that arose from archaic mtDNA, as individuals from these regions harbour both Neanderthal and Denisovan ancestry. Such NUMTs can be identified as a deviation from the actual mtDNA phylogeny in which modern humans form a monophyletic group compared to archaic humans [20]. We detected polymorphic mtDNA insertions in next generation sequencing data following the approach of Dayama et al. [8]. Afterwards we reconstructed sequences for these NUMTs, which were then analysed regarding their phylogeny, revealing the archaic ancestry of one NUMT. To discover the pathway of this NUMT into the modern human genome, we further analysed the flanking regions for archaic ancestry. Additionally, we detected population-specific patterns of polymorphic NUMTs which could be useful as markers in phylogenetic studies.

## Results

### Factors influencing the number of detected NUMTs

To detect polymorphic NUMTs, genomes were scanned for read pairs mapping both to chromosomal DNA and mtDNA as described in [8]. A total of 221 genomes from Indonesia and Oceania, from three studies, the Indonesian Genome Diversity Project (IGDP) [28], Simons Genome Diversity Project (SGDP) [29] and Vernot et al. [26] were analysed. A summary of the results for each study is shown in Table 1. In total, 1222 distinct NUMTs not annotated in the human reference genome were found (Additional file 2), with 1197 not detected in the 1000 Genomes Project (1000 GP) [30] and Human Genome Diversity Project (HGDP) samples analysed previously by Dayama et al. [8]. The 25 NUMTs already detected by Dayama et al. [8] were all present in samples from Europe, East Asia, America and Sub-Saharan Africa. The majority of NUMTs found in one study were not found in either of the other studies. These differences in NUMT patterns, even between populations within Oceania, supports previous observations of ongoing NUMT insertions in humans [4]. On average, 16.3 NUMTs were detected in each sample from the three high-coverage studies, compared to the average of 1.5 per sample found in the low to medium-coverage 1000 GP dataset. In addition, the ratio of distinct NUMTs to the total number found is very low in the 1000 GP dataset compared to others. Higher coverage thus seems to strongly increase the detection rate of this approach.

**Table 1:** Summary of discovered NUMTs in the different studies analysed; results for the 1000 GP dataset are taken from Dayama et al. [8]

Study	1000 GP [8]	SGDP (Oceanian samples)	Vernot et al.	IGDP
Populations	20	8	13	27
Analysed samples	946 (1000 GP) 53 (HGDP)	25	35	161
Coverage	∼4−6 (1000 GP) ∼5−20 (HGDP)	∼30−40	∼30−40	∼30−40
NUMTs per sample	∼1.5	16.0 ±6	13.4 ±3.3	16.8 ±10.2
Total NUMTs	∼1499	399	470	4035
Distinct NUMTs	141	171	121	1062
Study-specific NUMTs	115	128	81	975

Distinct NUMTs: amount of different NUMTs found in each study.

Study-specific NUMTs: amount of distinct NUMTs not found in any other study

**Fig. 2:**
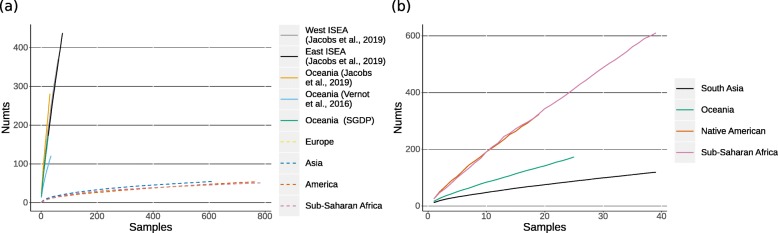
Comparison of rarefaction curves. Rarefaction curves plotted as the mean out of 100 repetitions. **a** Results from Island South East Asia (ISEA) and Oceania (continuous lines) are compared with worldwide 1000 GP data (dashed lines). **b** Comparison of different geographic regions within the Simons Genome Diversity Project (SGDP) dataset

We further analysed differences in NUMT diversity between different studies and larger geographical regions, using rarefaction analysis. Figure 2a shows a comparison of rarefaction curves for the different studies. For the 1000 GP dataset the rarefaction curves saturate at a low level, compared to the curves for the high-coverage studies, which do not reach saturation. Coverage therefore has a significant impact on the discovery of NUMTs. We also investigated NUMT diversity in different geographic regions, controlling for coverage by focusing on samples from the SGDP study. NUMT diversity here is higher in sub-Saharan Africa than in Oceania and South Asia (Fig. 2b). Additionally, we downsampled high coverage genomes to lower coverages and screened them for NUMTs. This analysis showed a moderate correlation between coverage and the amount of NUMTs detected (*r*^2^=0.35,*p*≤2.2*e*^−19^, Additional file 1: Figure S1), suggesting that many NUMTs may be missed in genomes with a coverage below 10.

### Ancestral and archaic NUMTs in Indonesian and Oceanian genomes

A sequence for each NUMT was reconstructed from the supporting reads. Those with a total sequence length below 20 base pairs (bp) were discarded. This cutoff was chosen to keep sequences that might contain enough phylogenetic information for further classification, but still filter out sequences that can not be classified. In most cases, one single sequence was generated. For some, multiple fragments were obtained and concatenated. These NUMTs were too long to be covered completely by short split reads or did not have sufficient read coverage in all positions. From one side of an insertion, a split read pair could span a part of the NUMT with a length of the sum of the mitochondrial read length, plus the insert size and the length of the clipped half of the chromosomal read. With an average read length of 150 bp and a median insert size up to 400 bp in the analysed samples, no single fragment longer than 1250 bp would be expected. The length of the fragments generated ranged between 26 and 774 bp (median: 70 bp), with the majority being shorter than 100 bp (Additional file 1: Figure S2a). For the high-coverage genomes used in our study, NUMTs with an overall mean coverage below 5x might result from sequencing artefacts. Sequences generated from such a low coverage are also more likely to be influenced by sequencing errors and therefore were discarded. As the coverage distribution for the NUMTs (up to 59x, median 14x, Additional file 1: Figure S2b) was not different from the rest of the genome sequence, filtering for excessive coverage was not required. GC-content for the NUMTs varied between 0.27 and 0.64 (median: 0.46) (Additional file 1: Figure S2c), similar to the the GC-content for the mtDNA genome (0.45), so no filtering based on GC-content was applied.

**Fig. 3:**
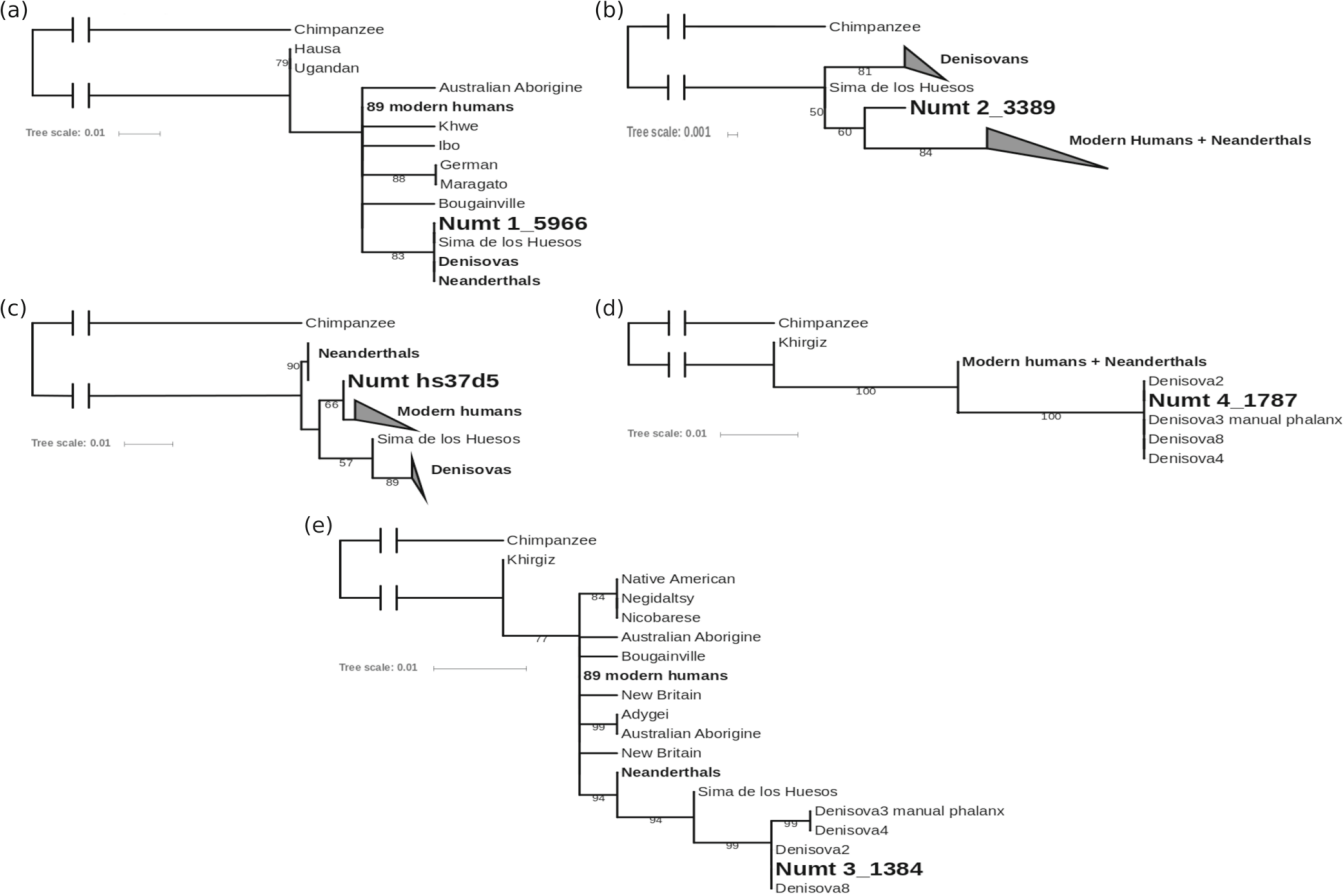
Pairwise nucleotide distances between NUMTs and mtDNA. Pairwise nucleotide distances vs. frequency (in logarithmic scale) within and between 97 modern humans (MHU, purple), 17 Neanderthals (NEA, blue), four Denisovans (DEN, green) and a specific NUMT (NUM, empty bars) for two ancestral NUMTs **a**, **b** and one Denisovan NUMT **c**

After filtering, a total of 2041 assembled fragments were obtained from all samples combined. These fragments belong to 172 distinct NUMTs. For each of these fragments the phylogeny was analysed by building a tree with the corresponding mtDNA fragments from various humans and hominins, using chimpanzee as an outgroup (Fig. 3).

**Fig. 4:**
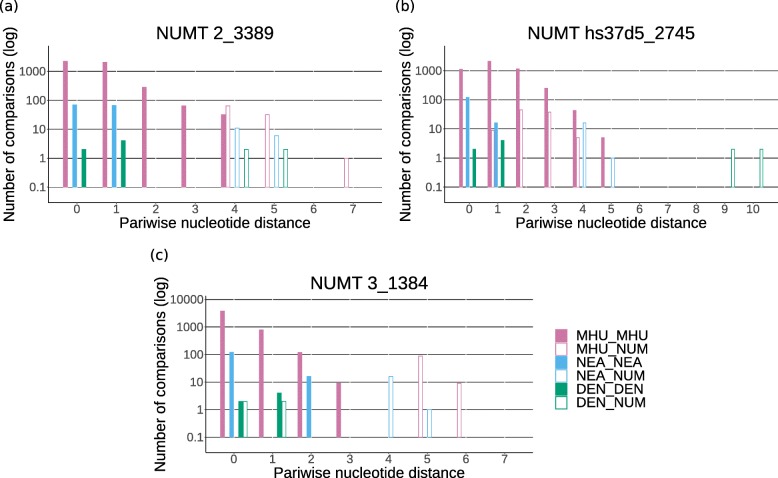
Phylogenetic trees for NUMTs. Maximum likelihood trees for putative ancestral NUMTs (**a**, **b**, **c**) and putative Denisovan NUMTs (**d**, **e**) in relationship with other hominin mtDNA sequences using distances based on nucleotide substitution rates. Ancestral NUMTs form a sister clade to at least all modern humans and are present in 1000 Genomes Project (1000 GP) samples from around the world, except for (**c**). Denisovan NUMTs are more similar to Denisovan mtDNA than to modern human mtDNA and are not present in 1000 GP samples. Bootstrap values over 50 are indicated at branch locations

The rooted trees were used to infer the origins of the NUMTs according to where their sequences fell within the hominin mtDNA phylogeny. To be able to classify a NUMT as either archaic, modern human or ancestral, at least some phylogenetic information is required, but the partial mtDNA sequences of some of the NUMTs are too short or conserved for accurate placement on the tree. Therefore only those trees for which either Denisovans, Neanderthals, modern humans, or Neanderthals and modern humans formed a monophyletic clade were considered for further analysis. A tree with a monophyletic clade of Neanderthals and Denisovans was also allowed as they both might represent the ancestral state for a region where modern humans show derived alleles. Additionally, trees placing the NUMT outside of all humans were used to classify it as ancestral to all humans. As this study aimed to detect archaic NUMT insertions, only those classified as not arising from modern humans were further analysed. To exclude that these are still part of modern human variation, pairwise nucleotide distances within and between modern humans, Neanderthals and Denisovans were calculated as in Fig. 4. For 98 NUMTs no useful tree could be generated. Either the alignments did not contain more than four distinct sequences or the trees did not contain any reasonable clades, as the sequences were too short or from mtDNA regions that were too conserved to contain enough phylogenetic information.

Most of the remaining NUMTs could be classified as modern human mtDNA according to their trees. For five NUMTs, the trees suggest an origin other than modern human mtDNA. Out of these five, two were also found by Dayama et al. [8] and are present in genomes from sub-Saharan Africa, Europe, Asia and America. The corresponding names from Dayama et al. [8] are given in brackets below.

#### Putative ancestral NUMTs

For NUMT 1_5966 (Poly_NumtS_67) the 58 bp sequence is identical with all corresponding Neanderthal and Denisovan mtDNA sequences, but differs at only one position from most modern humans (Fig. 3a). Therefore it cannot be confidently classified as archaic mtDNA. Taking into account its presence in worldwide modern human genomes, this NUMT was likely inserted in an ancestor of all modern humans, possibly even before the split with archaic humans, and therefore might resemble the ancestral state of this mtDNA region (Additional file 1: Figure S3). NUMT 2_3389 (Poly_NumtS_1239) forms a sister clade to all modern humans and Neanderthals (Fig. 3b). The 246 bp sequence inferred here is identical to that obtained by Dayama et al. [8] through Sanger sequencing. It is equally distant to modern humans, Neanderthals and Denisovans, but does not completely fall outside of modern human variation (Fig. 4a). Based on the comparison with an inferred ancestral human mtDNA, its age of insertion is estimated to be around 720,000 years ago, although this comparison does not allow a precise estimation [8]. This estimation falls into the proposed time range of the population split between archaic and modern humans of between 550,000 and 765,000 years ago [31] (Additional file 1: Figure S3). The presence of these two NUMTs in genomes from various populations around the world, including sub-Saharan Africa, suggests an insertion before the worldwide expansion of modern humans. NUMT hs37d5_2745 is 446 bp and was detected in the decoy sequences. These sequences are found in several *de novo* assemblies of the human genome, but are not present in the hg19 reference [32]. A BLAST search (https://blast.ncbi.nlm.nih.gov/Blast.cgi) for a 240 bp region flanking this NUMT in the National Center for Biotechnology Information database (https://www.ncbi.nlm.nih.gov/) showed the best hit for a human Bacterial Artificial Chromosome (BAC) clone from chromosome 4 (AC124864.3). The exact location of this BAC clone on chromosome 4 is not given, therefore the exact insertion site of the NUMT can not be localised. Its sequence forms a sister clade to all modern humans (Fig. 3c), but it does not fall outside of their variation (Fig. 4). The alignment contains only 15 positions with more than one allele in hominins. For one of these positions, the NUMT shares the ancestral state with chimpanzee and all archaic humans, whereas all modern humans share the derived allele (Additional file 1: Figure S4). This could indicate that the NUMT originated from an ancestral extinct or unsampled mtDNA lineage, but could also be due to a convergent mutation in the NUMT. Therefore it cannot be classified confidentially as ancestral.

#### Putative Denisovan NUMTs

The sequences for two NUMTs were identical to Denisovan mtDNA. NUMT 4_1787 was detected in five samples from west Indonesian populations speaking Austronesian languages (Additional file 1: Table S1) and is identical to the mtDNA sequence of all Denisovan individuals (Fig. 3d). However, the sequence obtained is only 43 bp long and differs from most other humans at just one position; thus, it cannot be confidently identified as Denisovan mtDNA.

NUMT 3_1384 is present in 15 samples from eastern Indonesia and New Guinea (Additional file 1: Table S1). A sequence of 251 bp was generated, which is identical to two Denisovan mtDNAs. It forms a clade with Denisovans and Sima de los Huesos, distinct from all other humans (Fig. 3e) and falls outside of all modern human and Neanderthal variation (Fig. 4c). The alignment contains 13 variable positions within hominins (Additional file 3). For five of these positions, Denisovans and the NUMT share an allele which differs from all modern humans. This suggests that it originated from Denisovan mtDNA rather than from mtDNA of a modern human or an ancestor of Denisovans and modern humans (Additional file 1: Figure S3). The phase of this NUMT was inferred in each sample using five phased genotypes (Additional file 1: Table S2).

### A Denisovan NUMT introgressed as nuclear DNA

**Fig. 5:**
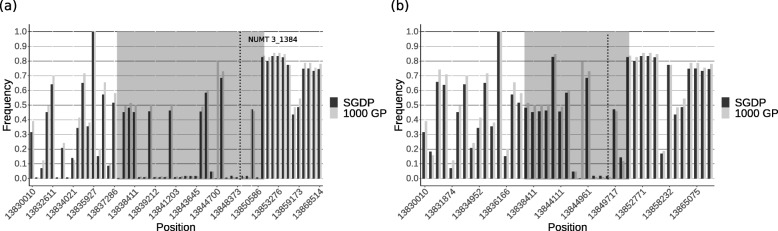
Worldwide frequencies of alleles shared with the Denisovan genome. Worldwide frequencies of alleles shared with the Denisovan genome in the region flanking NUMT 3_1384 in (**a**) Oceanian sample UV925, and (**b**) the same region in the sub-Saharan African sample NA19309. The alleles are within 20 kbp before or after the insertion site (vertical dashed line) and on the same phase. Frequencies were calculated in the 1000 Genomes Project (1000 GP) (black) and Simons Genome Diversity Project (SGDP) (grey) datasets. X-axis intervals are not linear, but indicate positions of shared alleles. High-frequency shared alleles reflect either homoplasy or incomplete lineage sorting; the greater abundance of low-frequency alleles shared with the Denisovan genome close to the insertion point (grey area) in the Oceanian sample vs. the sub-Saharan African sample suggests a Denisovan-introgressed haplotype in the Oceanian

To determine if the archaic NUMT introgressed within Denisovan nuclear DNA as explained in Fig. 1, the flanking regions were analysed for Denisovan ancestry using phased genotype data. For NUMT 3_1384, alleles shared between modern humans and Denisovans were identified in the flanking region. These alleles could be shared due to an introgression event, incomplete lineage sorting or homoplasy. As Denisovan ancestry is low or absent in populations outside of Oceania [23, 27], any Denisovan alleles in these populations presumably reflect the latter two cases, and hence can be used to distinguish them from true introgression. Figure 5a shows the frequencies of alleles in the SGDP and 1000 GP dataset shared between a Denisovan and an Oceanian sample containing NUMT 3_1384. Shared alleles with low frequencies in these worldwide datasets are abundant before and around the NUMT, suggesting the presence of an introgressed haplotype. Shared alleles further away from the insertion point mostly show higher frequencies in the worldwide datasets, suggesting that these alleles are either shared due to incomplete lineage sorting or homoplasy. Similar distributions of low and high frequency shared alleles were observed for all haplotypes containing NUMT 3_1384, indicating the end of that putatively introgressed haplotype around position chr3:13851000. In contrast, Fig. 5b shows the frequencies of shared alleles in the same region for a sample from sub-Saharan Africa, containing very few low-frequency shared alleles.

**Fig. 6:**
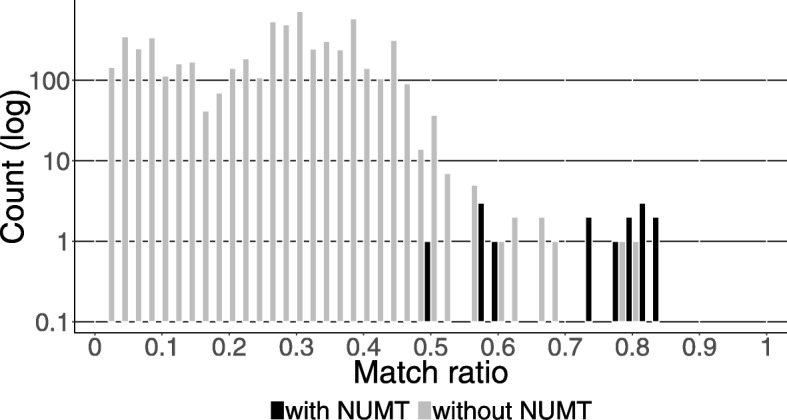
Match ratios of modern human genomes with the Denisovan Genome. Distributions of match ratios between modern humans and Denisovans for a 20 kbp region around NUMT 3_1384 in all samples from the 1000 GP, SGDP, IGDP and Verot et al. [26]. Match ratios were calculated for each phase individually counting shared non-reference alleles. Black bars represent haplotypes containing the NUMT insertion, grey bars represent haplotypes without the NUMT insertion

For the region around the insertion site of NUMT 3_1384, match ratios with the Denisovan genome were calculated as described above. Introgressed haplotypes are expected to show a higher match ratio than non-introgressed haplotypes. Due to the absence of Denisovan ancestry in European and sub-Saharan African populations, these populations can be used to estimate the expected distribution of match ratios for the haplotype of interest due to incomplete lineage sorting or chance; values higher than expected from this distribution suggest Denisovan ancestry. On average, 53 phased sites contained a non-reference allele, and the distribution of match ratios is shown in Fig. 6 (min = 0, Q1 = 0.13, mean = 0.25, Q3 = 0.35, max = 0.83,). Match ratios for haplotypes containing NUMT 3_1384 range from 0.59 to 0.83 with the exception of sample S_Papuan-5 (0.48). Except for this sample, they represent the highest 0.4% match ratios together with eight other haplotypes from two Europeans, two Southeast Asians, three Oceanians and one Indonesian. The regions flanking NUMT 3_1384 show higher similarity with the Denisovan genome than the same regions in all other analysed samples, suggesting a Denisovan origin of these flanking regions. The exception for sample S_Papuan-5 may reflect depletion of Denisovan ancestry through recent recombination around the insertion site.

For samples containing a heterozygous Denisovan NUMT insertion, the average match ratio of haplotypes containing the insertion (mean = 0.74) was significantly higher (one tailed paired t-test, p = 2∗10^−9^) than the average match ratio of haplotypes lacking the insertion (mean = 0.25). This further suggests that the NUMT insertion is part of a haplotype introgressed from Denisovans, whereas haplotypes for the same region but lacking the NUMT are not derived from Denisovans.

### The reference genome influences the detection of archaic NUMTs

NUMTs originating from Denisovan mtDNA might not be detected in genomes mapped against modern human mtDNA due to their diverged sequences. To test the effect of this potential reference bias, we remapped ten samples from Vernot et al. [26] (Additional file 1: Table S3) against a reference genome containing Denisovan mtDNA instead of modern human mtDNA. No additional NUMTs were detected. On average only 1.9 NUMTs were detected per sample, many fewer than the 16 NUMTs per sample in the original mapping files (Table 1). Calculating the coverage along the Denisovan reference mtDNA reveals gaps where only few or no modern human reads could be aligned (Additional file 1: Figure S5). Many modern human reads containing mtDNA sequences could not be aligned to the Denisovan reference, which reduces the number of detectable NUMTs. This also suggests that some Denisovan NUMTs might not be detected due to the use of a modern human reference for all analysed samples; however the regions that differ greatly between Denisovan and modern human mtDNA are short enough that reliable detection of NUMTs involving only those regions would be difficult anyway.

### Short read length complicates the identification of archaic NUMTs

Read pairs originating from NUMTs longer than 600 bp might not overlap the insertion site. Such read pairs will not be detected by our approach, but might contain important phylogenetic information. Therefore we analysed reads overlapping diagnostic mtDNA positions for different hominin lineages from Meyer at al. [31]. For the Neanderthal and Denisovan lineages, we could not detect more than five reads supporting the same diagnostic allele. For the Sima de los Huesos lineage, five reads supported a diagnostic allele at position 13391 in sample UV1134. An average of seven diagnostic positions for the Denisovan-Sima de los Huesos lineage were supported by 5-47 reads in each sample. This range of coverage is closer to the range of the nuclear chromosomal coverage of up to 50 x than to the mitochondrial coverage of around 1000 x.

**Fig. 7:**
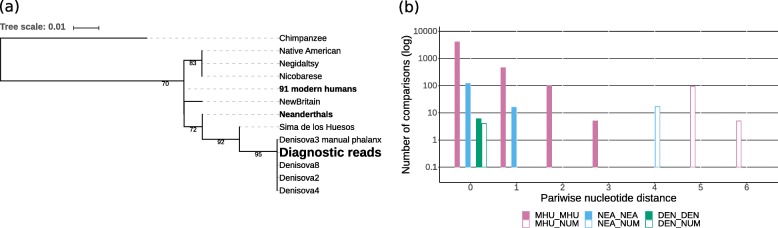
Phylogenetic analysis of diagnostic reads. A potential Denisovan NUMT inferred from diagnostic alleles. **a** Maximum likelihood tree for a 142 bp sequence generated from reads that contain the diagnostic allele for the Denisovan-Sima de los Huesos branch at position 9884. Bootstrap values over 50 are indicated at branch locations. **b** Pairwise nucleotide distances vs. frequency (in logarithmic scale) within and between 97 modern humans (MHU, purple), 17 Neanderthals (NEA, blue), four Denisovans (DEN, green) and a nuclear insert of mitochondrial DNA (NUMT) (NUM, empty bars)

For some of these positions, the sequence generated for the surrounding region falls outside of modern human variation and shows a phylogeny more similar to Denisovans and Sima de los Huesos than to modern humans (Fig. 7). This phylogeny, and the fact that the coverage is similar to the chromosomal coverage, suggests that these reads might originate from NUMTs that introgressed from an archaic hominin. They might belong to a detected NUMT which is too long to be covered by split reads, and the end of the NUMT might consist of conserved mtDNA regions that therefore do not distinguish this NUMT from NUMTs arising from other hominins.

## Discussion

In the human reference genome 755 NUMTs are annotated [7]. Most of these are fixed, with only 14 polymorphic in modern humans [16]. Our study focused solely on polymorphic insertions not present in the reference, using an approach that detects NUMTs in next generation sequencing data by analysing split reads [8]. We were able to discover a variety of polymorphic NUMTs in Indonesian and Oceanian genomes. Of these NUMTs, only 25 are also present in the 1000 GP samples. In addition to the 741 fixed NUMTs in the reference genome, our results suggest the presence of ∼ 16 polymorphic NUMTs on average in each human genome. This number might be even higher, as *dinumt* is not able to detect insertions in highly repetitive genomic regions. Most of these polymorphic NUMTs were only found in one study (Table 1), and therefore seem to be population-specific, further supporting their use as phylogenetic markers in modern human populations [15, 16]. The NUMTs that are specific to only some populations are most likely to have inserted around or after modern human dispersal from sub-Saharan Africa. These results provide further evidence for the ongoing transfer of mtDNA into the human nuclear genome even during recent human history [4, 6, 8].

A previous study of NUMTs in low to medium-coverage genomes could not find a strong correlation between average depth and detection rate [8]; however we find that with high-coverage genomes there is a 10-fold increase in the number of detected NUMTs. This is presumably because coverage depth is not uniform for all positions across the genome, but follows a Poisson distribution [33]. To detect a NUMT at a certain position, *dinumt* needs a minimum of 5 clipped reads. For the high-coverage samples we used, more than 95% of the genome is expected to be covered sufficiently to detect a NUMT. For a medium coverage of around 10x this fraction would be around 80%, and for low coverage less than 50% [34]. For the 1000 GP dataset, the rarefaction curves in Fig. 2a show that analysing more samples in a population does not necessarily lead to the discovery of many more NUMTs. This could mean that for some NUMTs to be detected, more samples cannot compensate for the lack of coverage. Merging samples from one population might be a solution to obtain sufficient read support to detect more NUMTs. In addition, the populations analysed differ among studies and thus might also influence a comparison of NUMT diversities on a smaller scale. In the 1000 GP many individuals were sampled from a few populations. The SGDP contains many more populations from around the world but many fewer individuals per population, and the Indonesian Genome Diversity Project (IGDP) [28] and the study of Vernot et al. [26] both sample much more intensively over a more focused geographic scale.

We identified three putative ancestral polymorphic NUMTs, two of which were also detected by Dayama et al. [8]. Their presence in sub-Saharan Africa and other populations around the world suggests their insertion in an ancestor of all modern humans, rather than as a result of archaic introgression. Their sequences place them outside of modern humans; however, in one case this classification was based on a difference at only one position. Even for these older polymorphic insertions, exact classification is not always possible due to the lack of sequence variation. We found an additional 23 NUMTs which were also detected by Dayama et al. [8] in samples from each continental group of the 1000 GP and thus these might also be of ancestral origin. Most of these are either not classifiable or were classified as of modern human origin.

The identification of ancient and archaic mtDNA in modern human genomes is constrained by two factors. On the one hand, conserved mtDNA is difficult to classify. On the other hand, strongly diverged sequences might not be detectable with the method we used. To be detected, their reads have to align to the mtDNA reference sequence, but if the NUMT sequence is too diverged from the reference, it might not align. Additionally, a read spanning the NUMT and the nuclear DNA has to be clipped, which will further decrease its mappability. Mapping approaches are in general optimized to map reads to a quite similar reference and not a highly diverged one. Changing the strategy by reducing the mismatch and clipping penalties might facilitate the mapping of such reads, and therefore enable more diverged sequences to be discovered [21, 35]. Another possibility would be to use another reference more similar to the target sequences. The remapping of some genomes to a reference genome containing Denisovan mtDNA instead of the revised Cambridge Reference Sequence (rCRS) demonstrates the impact of the reference on the detection of NUMTs. Although Denisovan and modern human mtDNA only show 3.5 mismatches on average for a read length of 150 bp [36], many modern human reads do not align to a Denisovan reference. No new NUMT could be detected, but the ability to detect modern human NUMTs strongly decreased. It might therefore be the case that there are more Denisovan NUMTs in the analysed genomes which could not be detected, even though we did not find any in 10 samples that were re-analysed with the Denisovan mtDNA as a reference. It could therefore be useful to explore the impact of reference bias in future studies.

Using short read sequencing technology restricts the maximum length for generated NUMT sequences to around 1,000 bp. Among the detected NUMTs, some were too long to be covered completely by split reads. For these we could only analyse the ends, which might not contain enough phylogenetic information to confidently classify them as to the hominin lineage of origin. By analysing reads which map to diagnostic positions, we were able to identify additional reads that might originate from Denisovan mtDNA. The presence of such reads suggests that there are additional Denisovan NUMTs present in the analysed genomes, but with our methods we could not confidently identify them as of Denisovan origin. These reads probably belong to longer NUMTs which are not fully covered by split reads. Conserved regions between the ends of these reads make it difficult to detect them, as they would also allow modern human reads to overlap. The advances in long-read sequencing technologies might enable the detection of such proposed long archaic NUMTs in the future.

Despite constraints in detecting NUMTs and the difficulties in classification of short mtDNA subsets, we were able to detect one NUMT that probably originated from Denisovan mtDNA. Its sequence contains five informative positions which classify it as Denisovan mtDNA, as opposed to originating from a common ancestor of modern humans and Denisovans. To further investigate how this NUMT ended up in a modern human genome, we considered two potential explanations. The first is that the insertion happened in a Denisovan individual (Fig. 1a) and that the NUMT later introgressed into modern humans within the nuclear Denisovan genome. Accordingly, the genomic region flanking the insert would also be introgressed and therefore should exhibit Denisovan ancestry. The other possibility is that a Denisovan female interbred with a modern human male (Fig. 1b). The maternal lineage of descendants of this interbreeding event would carry Denisovan mtDNA, from which a piece was inserted into the nuclear genome. Here, the flanking region could be of either modern human origin, or it could be an introgressed region, as the descendants of such an interbreeding event would also contain introgressed Denisovan nuclear DNA where such an insertion could happen. This becomes more and more unlikely with every subsequent interbreeding of this lineage’s offspring with modern humans, as the fragments of Denisova nuclear DNA would decline in length and number through segregation and recombination. Analysing the flanking region of the NUMT confidently classified as of Denisovan origin indicates a Denisovan ancestry. Although the second hypothesis cannot be fully rejected, it seems to be more likely, that the archaic NUMT is part of an introgressed haplotype.

Our results indicate that there are potentially many more NUMTs to be discovered via sequencing of additional populations to high coverage. Moreover, application of long-read technologies should also increase the number of detected NUMTs, promising to provide more insights into the introgression history of archaic and modern humans.

## Conclusions

We modified an existing method to detect NUMTs in next-generation sequence data, and applied the method to whole genome sequences from Indonesians and Papuans, in order to detect NUMTs arising from archaic human mtDNA. In high coverage genomes, an average of 16 NUMTs per individual is detectable. Most of these NUMTs seem to be population specific, indicating their insertion in recent human history. This finding further supports previous findings of an ongoing transfer of mtDNA to the nucleus in humans and suggests that the analysis of additional populations would lead to the discovery of many more NUMTs. A Denisovan NUMT could be identified in 16 samples from Indonesia and Oceania. Analyses of the flanking region of this NUMT reveals that it is part of a Denisovan haplotype. This suggests that the insertion of the NUMT most likely happened in a Denisovan individual and then introgressed into modern humans within nuclear DNA. Our pipeline can be applied to newly sequenced genomes in the future, which could reveal additional archaic NUMT insertions and new insights into the nature of interbreeding events.

## Methods

### Data analysis

To detect NUMTs, paired-end whole genome sequence reads aligned against the human reference genome version hg19 in BAM-format were used. In total, 221 Oceanian and Indonesian genomes were analysed for archaic NUMTs. 35 Papuan genomes with a median sequencing depth of 38 (min=33, Q1=35, Q3=39, max=43) were obtained from Vernot et al. [26], 25 Oceanian genomes with a median sequencing depth of 44 (min=34, Q1=42, Q3=45, max=51) from the SGDP [29] and 161 Indonesian and Papuan genomes with a median sequencing depth of 38 (min=18, Q1=35, median=38, Q3=43, max=48) from the IGDP [28] (Additional file 1: Table S4). For rarefaction analysis, an additional 40 sub-Saharan African, 19 native North American and 39 South Asian genomes from the SGDP were obtained (Additional file 1: Table S5). To determine the phase of the NUMTs and to analyse the ancestry of flanking regions, phased genotypes were obtained for all available samples from the 1000 GP [30], the SGDP, Vernot et al. [26] and the IGDP. If not mentioned otherwise, custom Python scripts were used to conduct all analyses. These scripts are available at https://github.com/robbueck/arcnumt.

### NUMT detection and analysis

For each sample, mean insert size and standard deviation of the read pairs were calculated using Picard CollectInsertSizeMetrics version: 2.17.10 (http://broadinstitute.github.io/picard/). NUMTs were detected using the *dinumt* software package [8]. The program detects NUMTs that are not annotated in the human reference genome by identifying read pairs where one end aligns to the mtDNA and its mate aligns to the nuclear genome. The amount of mismatches, gaps and clipping allowed depended on the mapping processes used by the three different studies. Each sample was analysed for NUMTs individually following the procedure described in Dayama et al. [8].

#### Rarefaction analysis and downsampling

Rarefaction curves were compared between studies to estimate the effect of coverage on the detection of NUMTs, and compared between regions within the SGDP dataset to estimate the difference between geographic regions. To determine if the number of detected NUMTs per genome had reached a threshold, which would indicate that increased sequencing would not reveal more NUMTs, NUMT rarefaction analysis was performed for each larger geographic region in each analysed dataset, including results for the 1000 GP dataset obtained from Dayama et al. [8]. Additional NUMT detection and rarefaction was done for all publicly-available sub-Saharan African, American and South Asian samples from the SGDP dataset (Additional file 1: Table S5). Samples were grouped into larger geographic regions, which were analysed individually for each study (SGDP: sub-Saharan Africa, America, Oceania, South Asia; 1000 GP: sub-Saharan Africa, America, Asia, Europe; IGDP: West Island South East Asia (ISEA), East ISEA, Oceania; Vernot et al. [26]: Oceania). For each group from each study, resampling was performed by successively adding all samples to the dataset. For each sample size, the number of different NUMTs in these samples were counted. The mean of 100 repetitions was calculated for each sample size and plotted in Fig. 2. A downsampling analysis was performed to further investigate the effect of coverage on the detection of NUMTS: the 25 Oceanian genomes from the SGDP were downsampled to lower coverages (Additional file 1: Figure S1) using samtools version 1.3.1 [37]. NUMT detection was performed as described above.

#### Reconstruction of NUMT sequences

A SAM-file containing the insertion supporting reads for each sample was obtained from *dinumt*. These reads were clustered according to the NUMT insertion they supported. As no NUMT insertions closer than 50 kbp were observed in our data, mitochondrial reads with mates mapping on the same chromosome within 2 kbp of each other were considered as supporting the same NUMT insertion and clustered together. The chromosome and the first four digits of the position of a NUMT were used to name it, e.g. a NUMT inserted at position 13848625 on chromosome 3 is named NUMT 3_1384. These mitochondrial reads were then mapped to the Reconstructed Sapiens Reference Sequence (RSRS) [38] of the mitochondrial genome, which represents the ancestral modern human mtDNA sequence, using BWA-MEM [39]. As the mitochondiral genome is circular, reads originating from the parts that are located at the beginning or the end of the reference genome would not map properly. Therefore nucleotide positions 1-1000 of the RSRS were copied and inserted after position 16569, to allow unbiased mapping to a circular genome. From the mapping output the RSRS coordinates for regions with a coverage of at least five reads were extracted. Positions with lower coverage were excluded to minimize the effect of sequencing errors. The NUMT sequence for these regions was obtained using GATKs HaplotypeCaller version 4.0.0 and FastaAlternativeReferenceMaker version 3.8.0 [40]. HaplotypeCaller was used to call variants for the mapping output. For each NUMT within one sample no heterozygous alleles were called, thus enabling the construction of one unambiguous consensus sequence for each NUMT within one sample using FastaAlternativeReferenceMaker. For some NUMTs, the sequence was broken down to multiple parts as the NUMT was too long to be fully covered by short reads, or not enough read coverage was available for each position of the NUMTs. In these cases, multiple sequences were obtained for one NUMT, which were concatenated according to their order on the RSRS, taking into account the circular nature of mtDNA.

#### Phylogeny of NUMT insertions

An alignment of mitochondrial genomes of 87 present day modern humans, 17 Neanderthals, ten ancient modern humans, four Denisovans, the Sima de los Huesos fossil, chimpanzee, the rCRS and the RSRS (Additional file 1: Table S6) was produced using MUSCLE version 3.8 [41]. For each NUMT, the corresponding mtDNA region was extracted from the alignment by using the RSRS coordinates adjusted for gaps in the aligned RSRS. The NUMT sequence was aligned with the corresponding mtDNA regions using MUSCLE version 3.8 [41]. Alignments were cleaned by removing identical sequences. If two or more sequences were identical, only one copy was kept along with the taxonomic information of the removed sequences. For each cleaned alignment containing more than three sequences, trees were built using RAxML version 8.2 [42]. Pairwise nucleotide distances within and between modern humans, Neanderthals, Denisovans and the NUMTs were calculated. In each possible sequence pair, the number of sites where the two sequences differed from each other were counted. A graphical overview of the pipeline is shown in Additional file 1: Figure S6.

#### Determining the phase of NUMT insertions

We determined the position of NUMTs within available phased chromosomal data by analysing NUMT reads that covered informative heterozygous positions that flanked the insertion point. The genotypes and the NUMT-reads were visualized together using the IGV Browser [43, 44]. The phase of a genotype supported by more than two thirds of all reads, and at least three NUMT reads, was assumed to be the phase of the NUMT insertion.

### Flanking region analysis

The flanking regions of the Denisovan NUMT were analysed for Denisovan ancestry in each sample with the NUMT. Therefore phased genotype data was obtained for all samples analysed for archaic NUMTs and additionally for all available 1000 GP and SGDP samples. Each phase in each sample was separately compared with the published Denisovan genome sequence [21]. Within a window of 20 kbp before and after the insertion site, all positions where a modern human shares a non-reference allele with the Denisovan genome were identified.

To detect alleles shared with Denisovans due to introgression, we calculated the frequency of all shared alleles in the 1000 GP and SGDP datasets. Alleles with a frequency below 0.05 in at least one of the two studies were considered to be shared likely due to introgression. The distribution of these low-frequency alleles around the insertion site of an archaic NUMT was used to estimate the boundary of a potentially introgressed haplotype as shown in Fig. 5.

We further analysed for Denisovan ancestry using a match ratio. For this analysis in a 20 kbp region around the insertion site each phase for each sample in all four datasets was individually compared to the Denisovan genome. In total, 2973 phased genomes were analysed. Unphased sites were excluded from the analysis. A match ratio was calculated as the proportion of sites where a phased genome and Denisovan shared a non-reference allele, compared to all sites where the phased genome or Denisovan contained a non-reference allele. For samples which are heterozygous for a Denisovan NUMT insertion, the match ratios of both phased regions were compared using a one tailed paired t-test. Under the assumption that the NUMT is part of an introgressed haplotype, the phased region without the Denisovan NUMT should show a significantly lower match ratio than the phased region with the Denisovan NUMT.

### Evaluation of potential biases

To examine the influence of the reference genome on the detection of archaic NUMTs, ten Oceanian genomes (Additional file 1: Table S3) were mapped against the hg19 reference genome with the mtDNA replaced by the mtDNA of Denisova8 (accession number: KT780370.1) using BWA-MEM [39]. NUMT detection and analysis were performed as described above. For each mapping file, coverage per position along the mtDNA was calculated using GATK DepthOfCoverage version 3.8 [40].

### Analysis of diagnostic alleles

As an alternative approach for detecting NUMTs from archaic humans, we screened genomes for reads mapping to the mitochondrial genome containing alleles specific to different branches of the phylogenetic tree of hominin mtDNA sequences. Diagnostic alleles on the mitochondrial genome for the branches in the mtDNA phylogenetic tree of Neanderthals, Denisovans, the Sima de los Huesos fossile and Denisovans-Sima de los Huesos were obtained from Meyer et al. [31]. For all samples from Vernot et al. [26], the reads were remapped to the rCRS using BWA-MEM [39]. For each set of diagnostic alleles, a pileup was performed at the position of each allele. All reads containing a diagnostic allele with a minimum base quality of 15 were extracted. Positions where less than five reads supported the diagnostic allele were discarded. For the region around each remaining position, a consensus sequence was constructed using the extracted reads and its phylogeny was analysed as described above for NUMTs.

## Supplementary information

Additional file1 **Table S1.** Summary of Oceanian and Indonesian samples analysed for archaic NUMTs.
**Table S2.** Additional SGDP samples from Africa, America and south Asia used for rarefaction analysis.
**Table S3.** Sequences in the alignment used for phylogenetic analysis of the NUMTs.
**Table S4.** Samples from Vernot et al., 2016 mapped against a custom reference genome containing Denisovan mtDNA.
**Table S5.** Summary of samples containing a putative Denisovan NUMT.
**Table S6.** Results for determining the phase of NUMT 3_1384 in all containing samples.
**Figure S1.** Number of NUMTs detected in downsampled genomes.
**Figure S2.** Mean coverage (a), GC-content (b) and sequence length (c) for reconstructed NUMT sequences.
**Figure S3.** Schematic tree for the hominin mitochondrial genome with putative NUMT insertions.
**Figure S4.** Alignment of NUMT hs37d5_2745 with Denisovans, Neanderthals and modern humans.
**Figure S5.** Per base coverage along Denisovan (mtDNA).
**Figure S6.** Workflow for NUMT and flanking region analysis.

Additional file2 NUMTs detected in Indonesian and Oceanian samples. A VCF-file containing the insertion points of all NUMTs detected in Indonesian and Oceanian samples.

Additional file3 Alignment of NUMT 3_1384. Alignment of the Denisovan NUMT 3_1348 with 87 present day modern humans, 17 Neanderthals, ten ancient modern humans, four Denisovans, the Sima de los Huesos fossil, chimpanzee, the rCRS and the RSRS. GenBank accession numbers are give for each sample.

## Abbreviations

1000 GP: 1000 genome project
BAC: Bacterial artificial chromosome
bp: Base pairs
HGDP: Human genome diversity project
ISEA: Island South East Asia
mtDNA: Mitochondrial DNA
NUMT: Nuclear Mitochondiral DNA segment
rCRS: Revised Cambridge reference sequence
RSRS: Reconstructed sapiens reference sequence
SGDP: Simons genome diversity project
