# Correction to: Archaic mitochondrial DNA inserts in modern day nuclear genomes

**DOI:** 10.1186/s12864-020-6449-8

**Published:** 2020-01-17

**Authors:** Robert Bücking, Murray P. Cox, Georgi Hudjashov, Lauri Saag, Herawati Sudoyo, Mark Stoneking

**Affiliations:** 1 Department of Evolutionary Genetics, Max Planck Institute for Evolutionary Anthropology, Deutscher Platz 6, D04103 Leipzig, Germany; 2 School of Fundamental Sciences, Massey University, Palmerston North, 4442 New Zealand; 3 Institute of Genomics, University of Tartu, 51010 Tartu, Estonia; 4 Genome Diversity and Diseases Laboratory, Eijkman Institute for Molecular Biology, Jakarta, 10430 Indonesia; 5 Department of Medical Biology, Faculty of Medicine, University of Indonesia, Jakarta, 10430 Indonesia; 6 Sydney Medical School, University of Sydney, Sydney, NSW 2006 Australia

**Correction to: BMC Genomics**

**https://doi.org/s12864-019-6392-8**

Following the publication of this article [1], the authors reported that the captions of Figs. 3 and 4 were published in the incorrect order, whereby they mismatch with their corresponding images. The figures are reproduced in the correct sequence with the correct captions in this Correction article. The original article has been corrected.

**Fig. 3:**
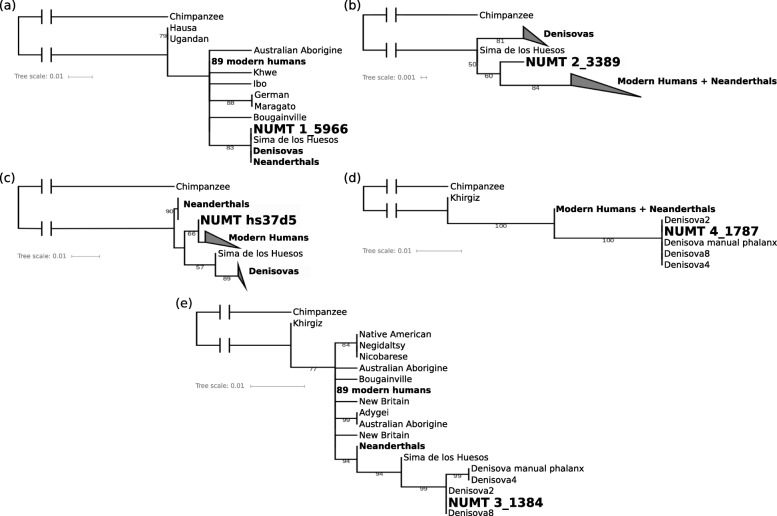
Phylogenetic trees for NUMTs. Maximum likelihood trees for putative ancestral NUMTs (**a**, **b**, **c**) and putative Denisovan NUMTs (**d**, **e**) in relationship with other hominin mtDNA sequences using distances based on nucleotide substitution rates. Ancestral NUMTs form a sister clade to at least all modern humans and are present in 1000 Genomes Project (1000 GP) samples from around the world, except for (**c**). Denisovan NUMTs are more similar to Denisovan mtDNA than to modern human mtDNA and are not present in 1000 GP samples. Bootstrap values over 50 are indicated at branch locations

**Fig. 4:**
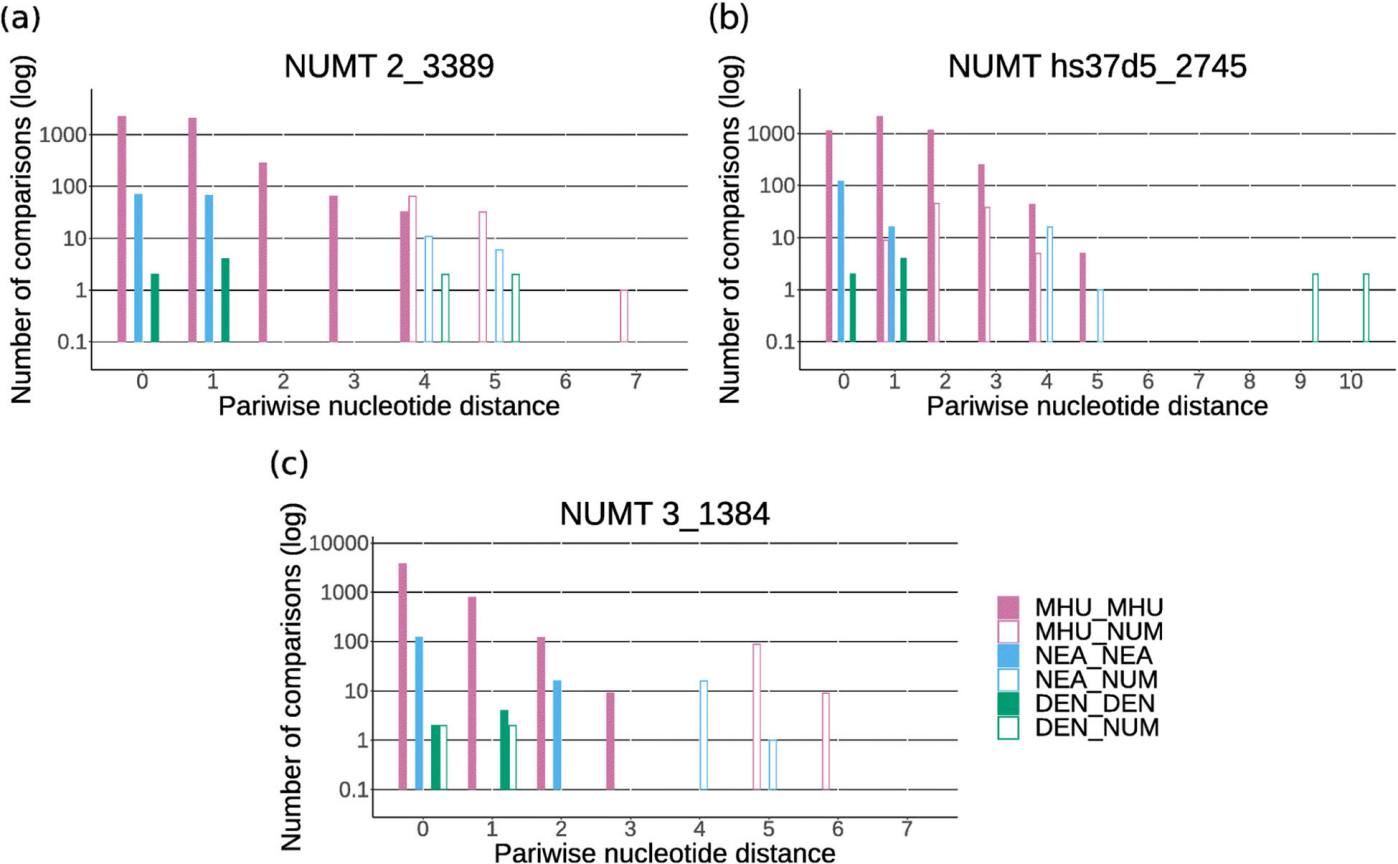
Pairwise nucleotide distances between NUMTs and mtDNA. Pairwise nucleotide distances vs. frequency (in logarithmic scale) within and between 97 modern humans (MHU, purple), 17 Neanderthals (NEA, blue), four Denisovans (DEN, green) and a specific NUMT (NUM, empty bars) for two ancestral NUMTs (**a**, **b**) and one Denisovan NUMT (**c**)
