# Supplementary material for: Archaic mitochondrial DNA inserts in modern day nuclear genomes
Source: BMC Genomics. 2019 Dec 26;20:1017. doi: 10.1186/s12864-019-6392-8 (PMC6933719; doi:10.1186/s12864-019-6392-8)
Supplement: Supplementary file 1 — Table S1. Summary of Oceanian and Indonesian samples analysed for archaic NUMTs. Table S2. Additional SGDP samples from Africa, America and south Asia used for rarefaction analysis. Table S3. Sequences in the alignment used for phylogenetic analysis of the NUMTs. Table S4. Samples from Vernot et al., 2016 mapped against a custom reference genome containing Denisovan mtDNA. Table S5. Summary of samples containing a putative Denisovan NUMT. Table S6. Results for determining the phase of NUMT 3_1384 in all containing samples. Figure S1. Number of NUMTs detected in downsampled genomes. Figure S2. Mean coverage (a), GC-content (b) and sequence length (c) for reconstructed NUMT sequences. Figure S3. Schematic tree for the hominin mitochondrial genome with putative NUMT insertions. Figure S4. Alignment of NUMT hs37d5_2745 with Denisovans, Neanderthals and modern humans. Figure S5. Per base coverage along Denisovan (mtDNA). Figure S6. Workflow for NUMT and flanking region analysis. [file 12864_2019_6392_MOESM1_ESM.pdf]

Supplement

Table S1: Summary of samples containing a putative Denisovan NUMT

NUMT	Sample	Country/Region	Population	Language classification
**4_1787**	NIAS17	Indonesia	Nias	Austronesian
	MTW078	Indonesia	Mentawai	Austronesian
	SMT01	Indonesian	Toba	Austronesian
	SMT23	Indonesia	Toba	Austronesian
	DNG17	Indonesia	Dieng	Austronesian
**3_1384**	UV030	Papua New Guinea	Baining	Papuan
	UV043	Papua New Guinea	Baining	Papuan
	UV1260	Papua New Guinea	Mangseng	Austronesian
	UV925	Papua New Guinea	Nakanai Bileki	Austronesian
	UV927	Papua New Guinea	Nakanai Bileki	Austronesian
	UV956	Papua New Guinea	Nakanai	Austronesian
	UV971	Papua New Guinea	Nakanai	Austronesian
	UV979	Papua New Guinea	Nakanai	Austronesian
	ALR06	Indonesia	Alor	Papuan
	ALR11	Indonesia	Alor	Papuan
	NG88	Papua New Guinea	?	Autronesian
	FAN-KEI-024	Indonesia	Faan	Austronesian
	S_Papuan-5	Papua New Guinea	?	?
	S_Papuan-10	Papua New Guinea	?	?

Table S2: Results for determining the phase of NUMT 3_1384 in all containing samples. The number of reads supporting a phase at one position are given in brackets, the number of reads supporting the other phase at a position are indicated as negative numbers.

NUMT	Sample	Positions				
		13848340	13848373	13848420	13848486	13848830
**3_1384**	UV030	homozygous	2 (11)	2 (12)	homozygous	homozygous
	UV043	homozygous	2 (4)	2 (8)	2 (14)	2 (7)
	UV1260	homozygous	homozygous	homozygous	homozygous	homozygous
	UV925	homozygous	2 (3)	2 (5)	2 (9)	2 (3)
	UV927	homozygous	2 (3)	2 (8)	2 (7)	2 (6)
	UV956	homozygous	1 (4)	1 (7)	homozygous	homozygous
	UV971	homozygous	2 (4)	2 (11)	2 (16)	2 (8)
	UV979	homozygous	2 (11)	2 (15)	2 (15)	2 (14, -1)
	ALR06	homozygous	2 (1)	2 (8)	2 (7)	2 (16)
	ALR11	homozygous	1 (1)	1(6)	homozygous	homozygous
	NG88	homozygous	2 (9)	2 (9)	2 (14)	2 (13, -1)
	FAN-KEI-024	1 (5)	1 (8)	1 (12)	homozygous	homozygous
	S_Papuan-5	homozygous	1 (7)	1 (9)	1 (9)	1 (8)
	S_Papuan-10	homozygous	1 (4)	1(4)	homozygous	homozygous

Table S3: Samples from Vernot et al., 2016 mapped against a custom reference genome containing Denisovan mtDNA

UV043, UV1003, UV1043, UV1134, UV1196, UV1224, UV1230, UV1260, UV1263, UV305

Table S4: Summary of Oceanian and Indonesian samples analysed for archaic NUMTs

Sample names	Country	Population	Location	Source
ALK-TBR-025	Indonesia	Alusi Krawain	Tanimbar	IGDP
ALR03 ALR04 ALR06 ALR11 ALR21	Indonesia	?	Alor	IGDP
BLI615	Indonesia	Gadon	Bali	IGDP
BNA01 BNA03-F BNA05 BNA12-F BNA14-F BNA21-F BNA22-F BNA25-F BNA26-F BNA29-F BNA32-F BNA40-F	Indonesia	Bena	Flores	IGDP
BRE05 BRE06 BRE10	Indonesia	Bere	Flores	IGDP
CBL002 CBL006 CBL010 CBL018 CBL019 CBL022 CBL025 CBL027 CBL054 CBL33 CBL34 CBL49 CBL55	Indonesia	Cibol	Flores	IGDP
DNG02 DNG05 DNG06 DNG09 DNG17 DNG32 DNG34	Indonesia	Dieng	Java	IGDP
DTT-KEI-006 DTT-KEI-031	Indonesia	Ohoidertutu	Kei	IGDP
FAN-KEI-011 FAN-KEI-024	Indonesia	Faan	Kei	IGDP
FDT-TBR-004	Indonesia	Fordata	Tanimbar	IGDP
FLT018 FLT020 FLT022 FLT042	Indonesia	Timur Kadakewa	Lembata	IGDP
FLT060 FLT077 FLT078	Indonesia	Waipukang	Lembata	IGDP
KJG02 KJG04 KJG08 KJG18 KJG25 KJG32	Indonesia	Kajang	Sulawesi	IGDP
MKT-TBR-007	Indonesia	Makatian	Tanimbar	IGDP
MPI-025 MPI-030 MPI-065 MPI-070 MPI-074 MPI-296 MPI-376	Indonesia	Mappi, Korowai	New Guinea	IGDP
MTW007 MTW020 MTW024 MTW030 MTW057 MTW058 MTW062 MTW066 MTW071 MTW078	Indonesia	?	Mentawai	IGDP
NG02-F NG06 NG09-F NG63-F NG65-F NG66 NG88-F	Papua New Guinea	Papua New Guinea	New Guinea	IGDP
NIAS01 NIAS02 NIAS03 NIAS04 NIAS08 NIAS10 NIAS12 NIAS13	Indonesia	Hilitobara	Nias	IGDP
NIAS15 NIAS16 NIAS17 NIAS21 NIAS26 NIAS33 NIAS38	Indonesia	Gomo	Nias	IGDP

RAM003 RAM005 RAM008 RAM022 RAM024 RAM025 RAM027-F RAM034-F RAM035-F RAM036-F RAM038 RAM039-F RAM041-F RAM043-F RAM045-F RAM046-F RAM067 RAM087 RAM105	Indonesia	Rampasasa	Flores	IGDP
SLD-TBR-001 SLD-TBR-022	Indonesia	Sangliat-Dol	Tanimbar	IGDP
SLW13 SLW36 SLW40 SLW42 SLW51 SLW54	Indonesia	Mandar	Sulawesi	IGDP
SMT01 SMT03 SMT05 SMT12 SMT22 SMT23 SMT39	Indonesia	Toba	Sumatra	IGDP
TL003 TL004 TL032 TL038 TL097 TL113 TL119	Indonesia	Ma'anyan	Borneo	IGDP
TMB-TBR-027	Indonesia	Tumbur	Tanimbar	IGDP
UV002 UV003 UV006 UV008 UV030 UV031 UV034 UV275B UV287B UV291 UV293 UV321B UV322B UV344B UV350 UV355B UV368B	Papua New Guinea	Baining	New Britain	IGDP
WAR-KEI-005 WAR-KEI-030	Indonesia	Waur	Kei	IGDP
B_Australian-3 B_Australian-4	Australia	Australian	sampling location unknown	SGDP
B_Papuan-15	Papua New Guinea	Papuan	sampling location unknown	SGDP
S_Bougainville-1 S_Bougainville-2	Papua New Guinea	Bougainville	Bougainville	SGDP
S_Dusun-1 S_Dusun-2	Brunei	Dusun	Tutong District	SGDP
S_Hawaiian-1	USA	Hawaiian	sampling location unknown	SGDP
S_Igorot-1 S_Igorot-2	Philippines	Igorot	Mountain Province, Luzon Island (sampled in Singapore)	SGDP
S_Maori-1	New Zealand	Maori	sampling location unknown	SGDP

S_Papuan-1 S_Papuan-10 S_Papuan-11 S_Papuan-12 S_Papuan-13 S_Papuan-14 S_Papuan-2 S_Papuan-3 S_Papuan-4 S_Papuan-5 S_Papuan-6 S_Papuan-7 S_Papuan-8 S_Papuan-9	Papua New Guinea	Papuan	New Guinea	SGDP
UV043 UV1266	Papua New Guinea	Baining	West New Britain	Vernot et al., 2016
UV1003	Papua New Guinea	Nakanai Loso	West New Britain	Vernot et al., 2016
UV1042 UV1043 UV1224	Papua New Guinea	Mamusi	West New Britain	Vernot et al., 2016
UV1134	Papua New Guinea	Ata	West New Britain	Vernot et al., 2016
UV1196	Papua New Guinea	Melamala	West New Britain	Vernot et al., 2016
UV1230	Papua New Guinea	Ata	West New Britain	Vernot et al., 2016
UV1260	Papua New Guinea	Mangseng	West New Britain	Vernot et al., 2016
UV1263	Papua New Guinea	Pasismanua	West New Britain	Vernot et al., 2016
UV305	Papua New Guinea	Lavongai	West New Britain	Vernot et al., 2016
UV500	Papua New Guinea	Mussau	West New Britain	Vernot et al., 2016
UV518 UV573	Papua New Guinea	Nailik	Mussau	Vernot et al., 2016
UV580 UV886 UV897 UV910 UV919 UV923 UV925 UV927 UV929 UV931 UV940	Papua New Guinea	Nakanai Bileki	New Ireland	Vernot et al., 2016
UV944 UV946 UV952 UV956 UV958 UV964 UV971 UV979 UV986	Papua New Guinea	Bileki	West New Britain	Vernot et al., 2016

Table S5: Additional SGDP samples from Africa, America and south Asia used for rarefaction analysis

**Africa:**
B_Dinka-3, B_Ju_hoan_North-4, B_Mandenka-3, B_Mbuti-4, B_Yoruba-3, S_BantuHerero-1, S_BantuKenya-1, S_BantuKenya-2, S_BantuTswana-1, S_BantuTswana-2, S_Dinka-1, S_Dinka-2, S_Esan-1, S_Esan-2, S_Gambian-1, S_Gambian-2, S_Ju_hoan_North-1, S_Ju_hoan_North-3, S_Khomani_San-1, S_Khomani_San-2, S_Luhya-1, S_Luhya-2, S_Luo-1, S_Luo-2, S_Mandenka-1, S_Mandenka-2, S_Masai-1, S_Masai-2, S_Mbuti-1, S_Mbuti-2, S_Mbuti-3, S_Mende-1, S_Mende-2, S_Mozabite-1, S_Mozabite-2, S_Saharawi-1, S_Saharawi-2, S_Yoruba-1, S_Yoruba-2
**America:**
B_Karitiana-3, B_Mixe-1, S_Chane-1, S_Karitiana-1, S_Karitiana-2, S_Mayan-2, S_Mixe-2, S_Mixe-3, S_Mixtec-1, S_Mixtec-2, S_Piapoco-2, S_Pima-1, S_Pima-2, S_Quechua-1, S_Quechua-2, S_Surui-1, S_Surui-2, S_Zapotec-1, S_Zapotec-2
**South Asia:**
S_Balochi-1, S_Balochi-2, S_Bengali-1, S_Bengali-2, S_Brahmin-1, S_Brahmin-2, S_Brahui-1, S_Brahui-2, S_Burusho-1, S_Burusho-2, S_Hazara-1, S_Hazara-2, S_Irula-1, S_Irula-2, S_Kalash-1, S_Kalash-2, S_Kapu-1, S_Kapu-2, S_Khonda_Dora-1, S_Kusunda-1, S_Kusunda-2, S_Madiga-1, S_Madiga-2, S_Makrani-1, S_Makrani-2, S_Mala-2, S_Mala-3, S_Pathan-1, S_Pathan-2, S_Punjabi-1, S_Punjabi-2, S_Punjabi-3, S_Punjabi-4, S_Relli-1, S_Relli-2, S_Sindhi-1, S_Sindhi-2, S_Yadava-1, S_Yadava-2

Table S6: Sequences in the alignment used for phylogenetic analysis of the NUMTs.

**Present day modern humans:**
AY963585_Ugandan116569/116569, AF347009_San116566/116566, AF346968_Biaka116566/116566, AF346987_Ibo116565/116565, AF346996_Mbenzele116565/116565, AY195777_Khwe116557/116557, AY195783_San116567/116567, AF346976_Effik116569/116569, AF346995_Mandenka116567/116567, AF346985_Hausa116566/116566, AY956414_NewBritain116570/116570, AF347003_PNGCoast116569/116569, AY289081_PNGCoast116568/116568, AY289079_PNGCoast116569/116569, AY289089_PNGHigh116568/116568, AY289097_TaiwanAborigine116572/116572, DQ137403_NewBritain116569/116569, AF347000_Mkamba116568/116568, DQ137411_Bougainville116569/116569, AF346964_Aust116575/116575, AY519489_Negidaltsy116559/116559, AY570524_Mansi116571/116571, AF346971_Chukchi116566/116566, AY195760_Korea116567/116567, AY289073_Koraga116569/116569, AY882396_Adygei116568/116568, DQ137408_NewBritain116568/116568, AY195764_Caucasian116570/116570, AF347014_Yoruba116566/116566, AF381998_Jordan116566/116566, AY289067_Aust116571/116571, AY289091_PNGHigh116570/116570, AF347004_PNGHigh116571/116571, AY289053_Aust116570/116570, AF347012_Warao116565/116565, AY195759_NativeAmerican116565/116565, AY519490_Nganasan116568/116568, AY195772_Udegei116570/116570, AY519487_Koryak116568/116568, AY195790_NanajNegidal116570/116570, AF346994_Lisongo116568/116568, AY195749_NativeAmerican116563/116563, AF346966_India116568/116568, AY195791_Malay116567/116567, AY289086_PNGHigh116568/116568, AY950294_Onge116568/116568, AY289055_Aust116569/116569, AF346983_German116568/116568, AF382001_Maragato116567/116567, AY950291_Onge116568/116568, AY289092_PNGHigh116568/116568, AY195773_Finland116569/116569, AF346991_Khirgiz116557/116557, AJ842750_TaiwanAborigine116559/116559, AY195770_TofalarNegidal116560/116560, AJ842747_TaiwanAborigine116557/116557, AF347007_Samoa116560/116560, AY289069_Cook116559/116559, AY289083_PNGCoast116560/116560, AF346963_Aust116568/116568, AY289096_TaiwanAborigine116571/116571, AY289054_Aust116569/116569, AY950287_Nicobarese116557/116557, AY963580_Malay116568/116568, AY289088_PNGHigh116569/116569, AY882384_Adygei116570/116570, AY519484_Buriat116559/116559, AY275536_Berber116567/116567, AY289060_Aust116569/116569, AF382005_Leon116571/116571, AY882387_Italian116573/116573, AY289063_Aust116568/116568, AY289051_Aust116567/116567, AF381990_Berber116568/116568, AY275532_Maragato_Leon116569/116569, AY882398_Adygei116570/116570, AF347011_Uzbek116561/116561, AY289100_Thai116560/116560, AY275534_Morocco116570/116570, AY275535_Mauritania116571/116571, AF346974_Tatar116570/116570, AY195747_Caucasian116567/116567, AY275530_Galicia116569/116569, AY882412_Berber116571/116571, AY882400_Italian116570/116570, AY882404_Saami116569/116569, AF381997_Jordan116568/116568, rCRS_NC_012920116569/116569
**Ancient modern humans:**
Eskimo_Saqqaq_4000116569/116569 Iceman_4550116576/116576, BS11_8281116559/116559, Kostenki_37958116568/116568, DolniVestonice13_31488116570/116570, Loschbour_8054116568/116568, Oberkassel998_13518116570/116570, Tianyuan_40328116559/116559, DolniVestonice14_31488116569/116569, Ust_Ishim116570/116570
**Neanderthals:**

Neanderthal116566_GoyetQ571/116566 KX198082.1, Neanderthal_Okladnikov2_KF982693116566/116566, Neanderthal116565_GoyetQ573/116565 KX198083.1, Neanderthal116565_GoyetQ561/116565 KX198084.1, Neanderthal116566_GoyetQ572/116566 KX198088.1, Neanderthal_Feldhofer1_FM865407116565/116565, Neanderthal_Vindija33.25_FM865410116565/116565, Neanderthal116563/116563 GoyetQ3057 KX198086.1, Neanderthal116565_GoyetQ374a1/116565 KX198085.1, Neanderthal_Vindija33.16_AM948965116565/116565, Neanderthal_Vindija33.19_KJ533545116565/116565, Neanderthal_Vindija33.17_KJ533544116565/116565, Neanderthal_ElSidron1253_FM865409116565/116565, Neanderthal116565_GoyetQ3054/116565 KX198087.1, Neanderthal_Feldhofer2_FM865408116565/116565, Neanderthal_Altai_KC879692116567/116567, Neanderthal_Mezmaiskaya1_FM865411116565/116565
**Denisovans:**
Denisova3_manual_phalanx_NC_013993116570/116570, Denisova4_FR695060116570/116570, Denisova2116570/116570, Denisova8116573/116573
**Other:**
NC_001643.1_PanA1598616554/116554 troglodytes mitochondrion<br>KF683087.1_Homo_heidelbergensis<br>RSRS

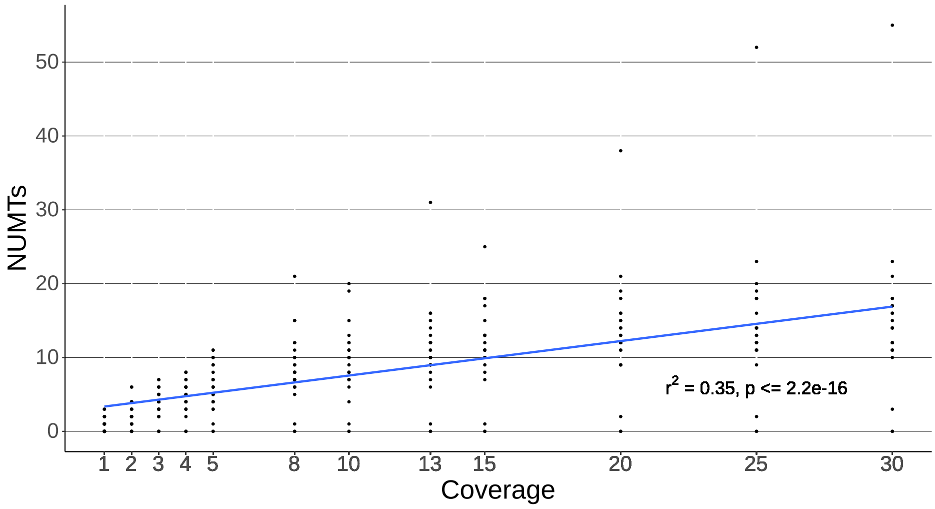

Figure S1: Number of NUMTs detected in genomes that were downsampled to a certain coverage. Each dot represents one of the 25 Oceanian genomes from the SGDP downsampled to each coverage. There is a modest significant correlation ($r^2 = 0.35, p \leq 2.2e^{-19}$) between coverage and the amount of discovered NUMTs.

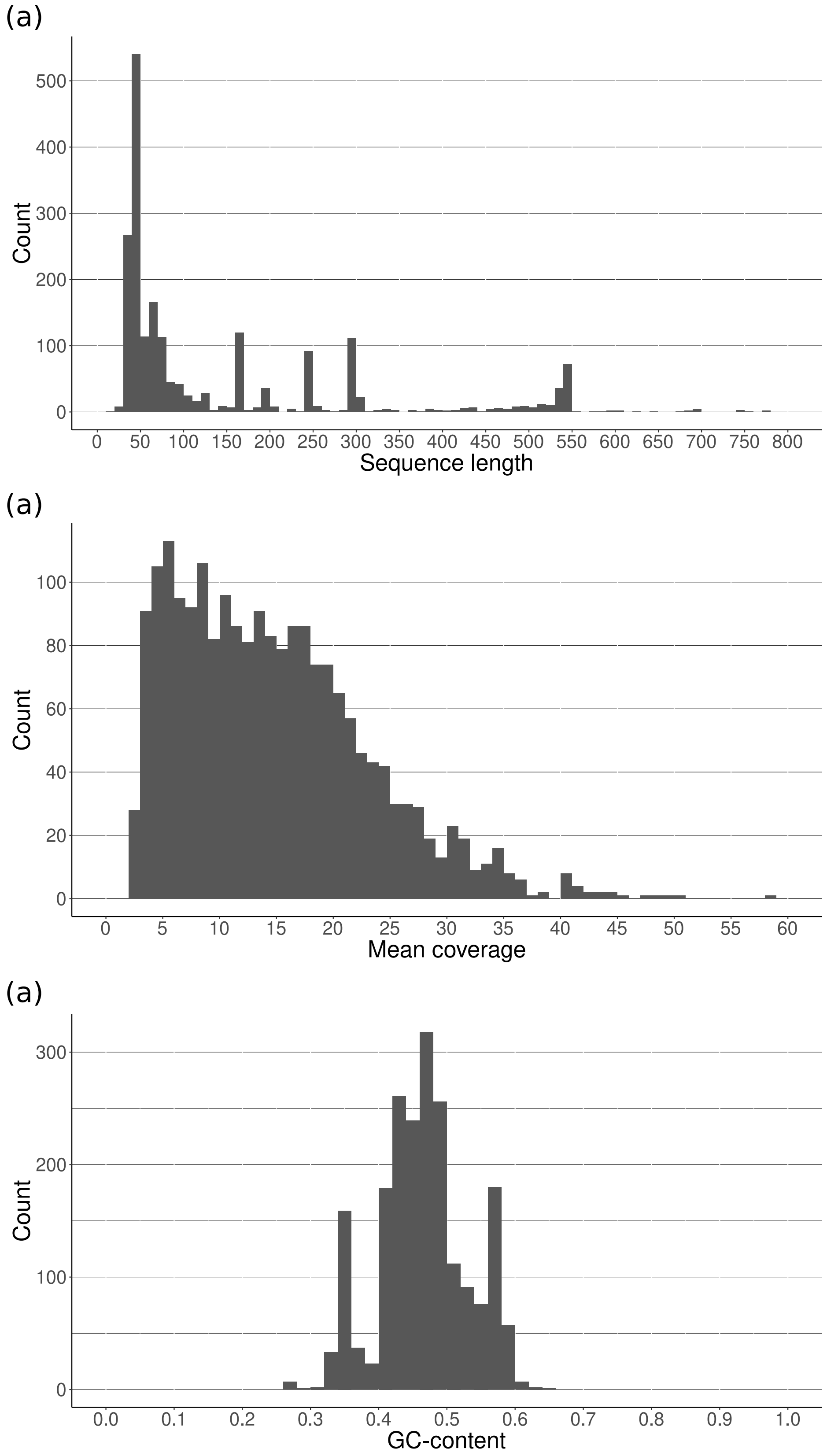

Figure S2: Mean coverage (a), GC-content (b) and sequence length (c) for reconstructed NUMT sequences.

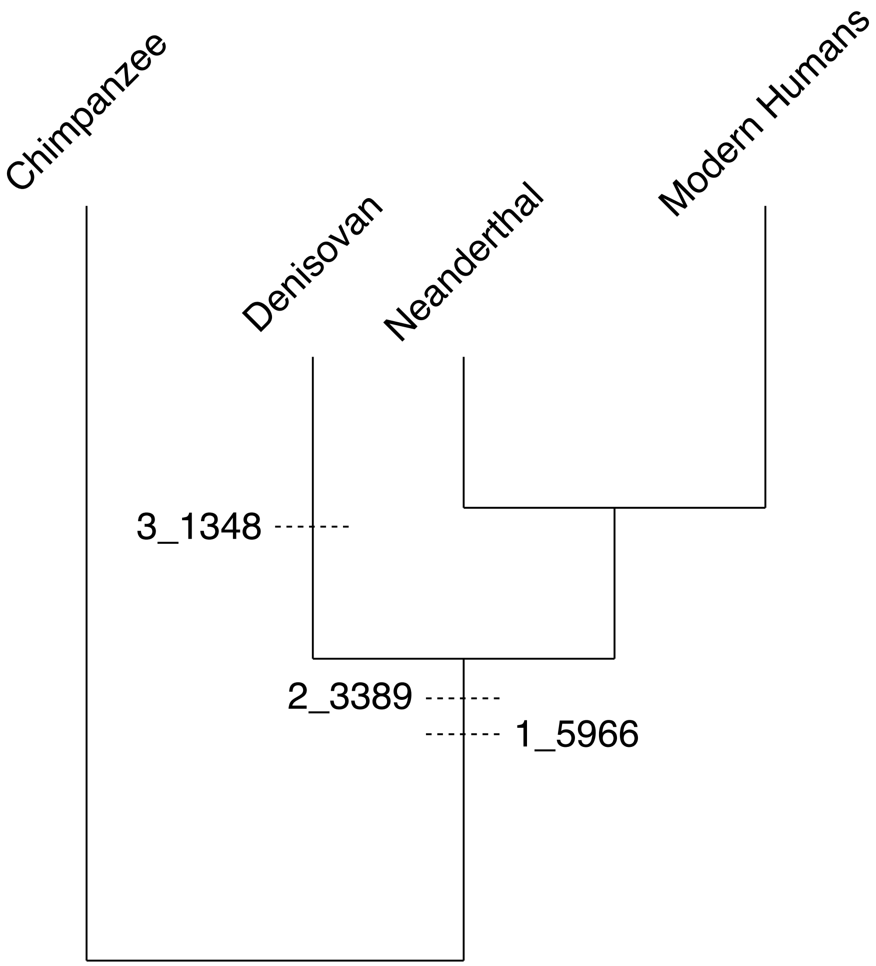

Figure S3: Schematic tree for the hominin mitochondrial genome. Vertical dashed lines indicate the lineage where ancestral and archaic NUMTs putatively originated from.

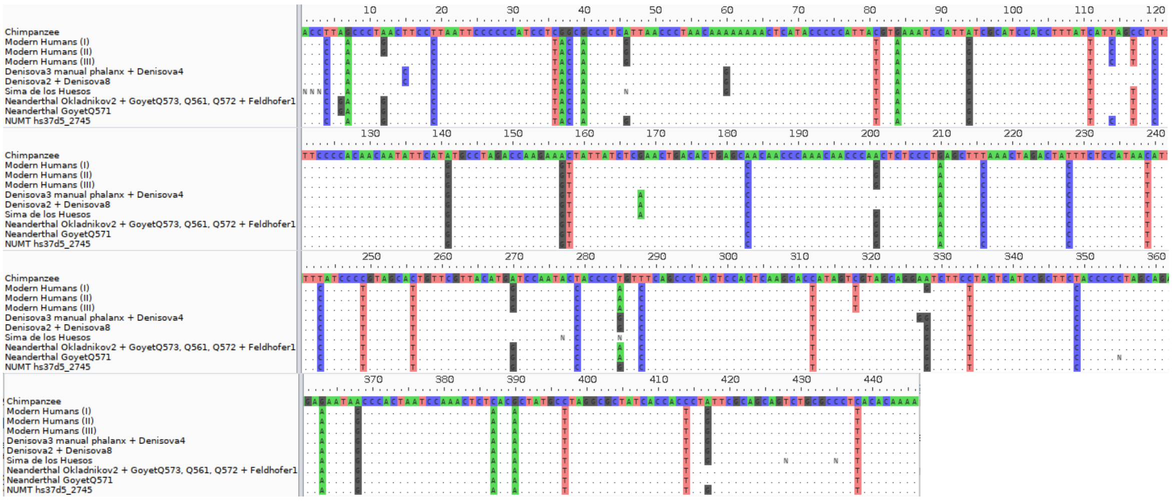

Figure S4: Alignment of NUMT hs37d5_2745 with Denisovans, Neanderthals and modern humans (I: Ugandan, San, Biaka, Ibo, Mbenzele, Khwe, Hausa, Yoruba, Jordan; II: Effik, Mandenka, NewBritain, PNGCoast, PNGHigh, NewBritain, Bougainville, Mansi, Chukchi, Korea, Indigenous Australian, Warao, Native American, Nganasan, Udegei, Koryak, Nanaj Negidal, Saqqaq, Lisongo, Onge, Finnland, Tofalar Negidal, Taiwan Aborigine, Samoa, Cook Island, Buriat, Berber, Uzbek, Thai, Tatar, Caucasian, Tianyuan 1, Ust Ishim; III: Caucasian, Adygei, Berber, Leon, Iceman, Italian, Morocco, Mauritania, Dolni Vestonice 13, Dolni Vestonice 14, Kostenki, Galicia, Loschbour, Oberkassel998).

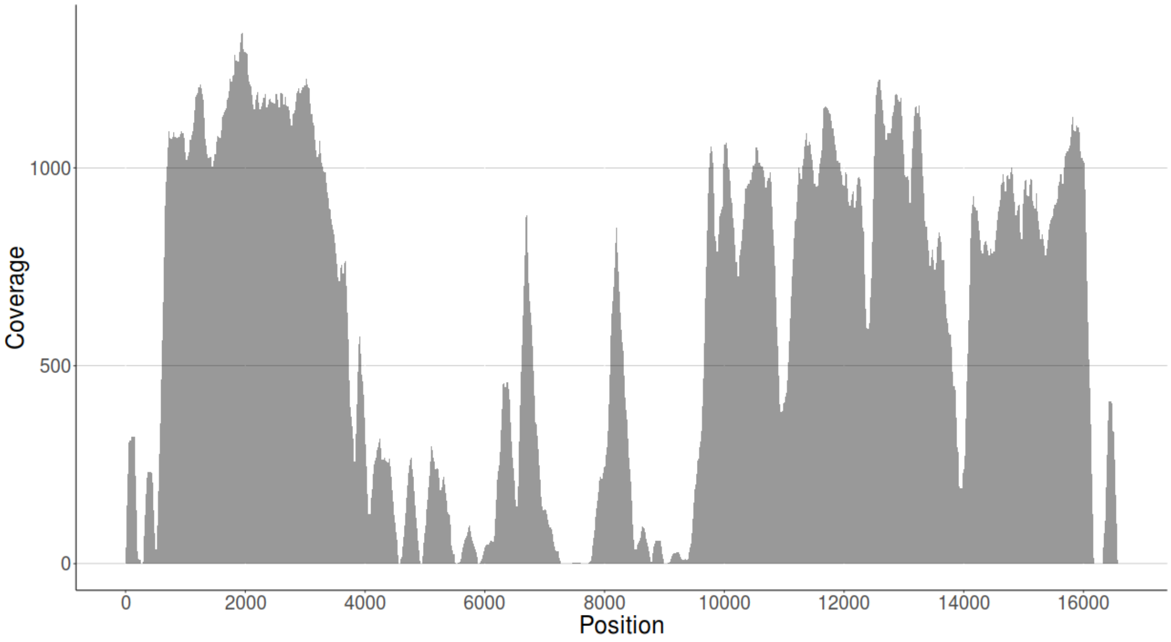

Figure S5: Per base coverage along mitochondrial DNA (mtDNA). The genome of a modern human was mapped to the hg19 reference genome with the mtDNA exchanged for Denisovan mtDNA.

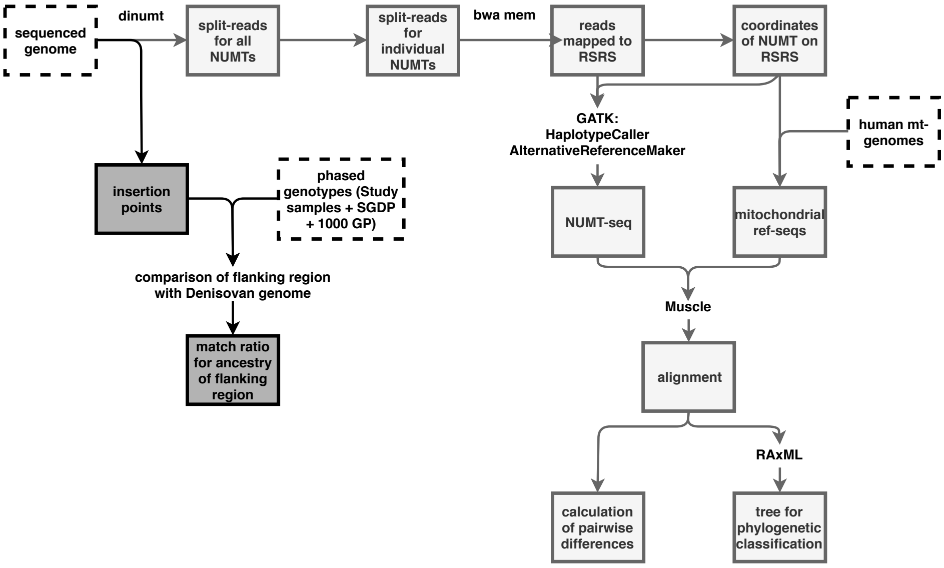

Figure S6: Workflow for NUMT and flanking region analysis.
